# Anti-Warburg effect by targeting HRD1-PFKP pathway may inhibit breast cancer progression

**DOI:** 10.1186/s12964-020-00679-7

**Published:** 2021-02-15

**Authors:** Ya Fan, Jia Wang, Yuemei Xu, Yipin Wang, Tao Song, Xiubin Liang, Feng Jin, Dongming Su

**Affiliations:** 1 Department of Pathology, Nanjing Medical University, Nanjing, Jiangsu People’s Republic of China; 2 Department of Breast Surgery, Institute of Breast Disease, The Second Hospital of Dalian Medical University, Dalian, Liaoning People’s Republic of China; 3 Department of Pathology, Nanjing Drum Tower Hospital, The Affiliated Hospital of Nanjing University Medical School, Nanjing, Jiangsu People’s Republic of China; 4 Center of Pathology and Clinical Laboratory, Sir Run Run Hospital of Nanjing Medical University, Nanjing, Jiangsu People’s Republic of China; 5 Department of Pathophysiology, Nanjing Medical University, Nanjing, Jiangsu People’s Republic of China; 6 Department of Breast Surgery, The First Affiliated Hospital of China Medical University, Shenyang, Liaoning Province People’s Republic of China

**Keywords:** Anti-warburg effect, Cancer progression, Molecular patterns, Treatment target

## Abstract

**Background:**

Our previous studies have shown that the E3 ubiquitin ligase of HMG-CoA reductase degradation 1 (HRD1) functions as a tumor suppressor, as overexpression of HRD1 suppressed breast cancer proliferation and invasion. However, its role in breast cancer cell glucose metabolism was unclear. Here, our aim was to uncover the role and molecular mechanisms of HRD1 in regulating aerobic glycolysis in breast cancer.

**Methods:**

The effect of HRD1 on robic glycolysis in breast cancer cells were assessed. Then the proliferation, colony formation ability, invasion and migration of breast cancer cells were evaluated. The relationship between HRD1 and PFKP was validated by Mass spectrometry analysis, immunofluorescence and co-immunoprecipitation. The level of PFKP ubiquitination was measured using ubiquitylation assay. Furthermore, the tumor growth and metastasis in mice xenografts were observed.

**Results:**

We found that upregulation of HRD1 clearly decreased aerobic glycolysis, and subsequently inhibited breast cancer proliferation and invasion. Mass spectrometry analysis results revealed a large HRD1 interactome, which included PFKP (platelet isoform of phosphofructokinase), a critical enzyme involved in the Warburg Effect in breast cancer. Mechanistically, HRD1 interacted and colocalized with PFKP in the cytoplasm, targeted PFKP for ubiquitination and degradation, and ultimately reduced PFKP expression and activity in breast cancer cells. HRD1 inhibited breast cancer growth and metastasis in vivo through a PFKP-dependent way

**Conclusions:**

Our findings reveal a new regulatory role of HRD1 in Warburg effect and provide a key contributor in breast cancer metabolism.

**Video abstract**

**Graphic abstract:**

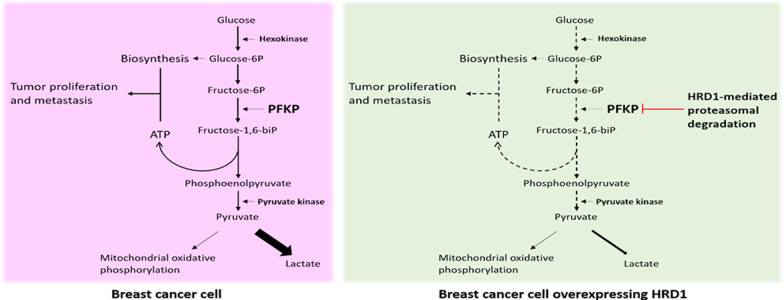

## Background

Breast cancer is the leading cause of cancer-related mortality among women worldwide due to metastasis, recurrence, or chemoresistance [1]. Breast cancer cell proliferation, survival, migration, and drug resistance depend on aerobic glycolysis (termed the Warburg Effect), a key feature of energy metabolism that converts glucose into lactate even in the presence of sufficient oxygen [2, 3]. Growing evidence now demonstrates that relieving the Warburg effect can be efficacious in inhibiting breast cancer proliferation and invasion [4, 5]. Therefore, a better understanding the regulatory mechanism of the Warburg Effect, and especially of the key glycolytic enzymes involved in aerobic glycolysis, has the potential to identify glycolysis-based therapies for breast cancer.

One glycolytic enzyme, the platelet isoform of phosphofructokinase (PFKP), catalyzes the irreversible conversion of fructose-6-phosphate to fructose-1,6-bisphosphate and is the first rate-limiting step in glycolysis. Breast cancer is characterized by high PFKP expression that, in turn, has been associated with decreased patient survival [6]. Accumulating evidence has demonstrated that PFKP plays a crucial role in promoting aerobic glycolysis and lactate production in breast cancer cells, thereby stimulating cancer cell proliferation and metastasis [7, 8]. Deletion of PFKP can significantly inhibit lactate production to impair invasion and migration by breast cancer cells [9, 10]. Therefore, suppression of PFKP expression and activity represents a viable strategy for breast cancer treatment.

The expression and activity of PFKP are mainly regulated through post-translational modifications such as acetylation, phosphorylation, and ubiquitination [11]. The PFKP molecule contains 12 acetylation sites and 18 phosphorylation sites, and strong interactions have been demonstrated between acetylation and phosphorylation.

Acetylation of PFKP at K395 by lysine acetyltransferase 5 triggers its transport to the plasma membrane and phosphorylation at Y64. This, in turn, leads to the activation of AKT, which increases PFKP activity by the production of fructose-2,6-bis-phosphate and elevates PFKP expression by enhancing PFKP stability [12]. In glioblastomas, PFKP S386 phosphorylation prevents the TRIM21-mediated ubiquitylation and degradation of PFKP, leading to an increase in aerobic glycolysis [13]. However, the mechanism underlying the regulation of PFKP stability in breast cancer is unknown.

One possible regulator is HMG-CoA reductase degradation protein 1 (HRD1), also known as synoviolin. This is a RING finger domain-containing E3 ubiquitin ligase that plays an important role in tumorigenesis and tumor progression [14–16]. HRD1 acts an E3 ligase, with biological roles closely related to its substrates. Our previous study demonstrated that HRD1 inhibited the growth and metastasis of breast cancer cells by targeting IGF-1R and that it sensitized breast cancer cells to tamoxifen by targeting S100A8 for ubiquitylation and degradation [17–19]. In the current study, we used mass spectrometry analysis to identify an interaction between PFKP and HRD1 in MDA-MB-231 cells. Further study showed that HRD1 catalyzed PFKP ubiquitination and promoted PFKP degradation, indicating a potential involvement of HRD1 in aerobic glycolysis in breast cancer cells.

## Materials and methods

### Cell lines and antibodies

Human breast cancer (MCF-7, MDA-MB-231) and embryonic kidney 293 T (HEK293T) cell lines were cultured as previously reported [17]. The HRD1 antibody for western blotting was obtained from Sigma Aldrich (St. Louis, MO, USA). PFKP antibody was obtained from Cell Signaling Technology. HRD1 antibody for IHC was purchased from Abgent (San Diego, California, USA). Antibodies against β-Actin, myc-Tag, and HA-Tag were acquired from Santa Cruz Biotechnology (Santa Cruz, CA, USA).

### Seahorse assay

The extracellular acidification rate (ECAR) and oxygen consumption rate (OCR) were measured using a Seahorse XF96 Flux Analyzer (Seahorse Bioscience, Billerica, Massachusetts, USA). MCF-7 and MDA-MB-231 cells (2 × 10^4^/well) were seeded into an XF96-well plate and allowed to attach overnight. For ECAR assay, cells were pre-incubated with unbuffered media for 1 h, followed by a sequential injection of 10 mmol/L glucose, 1 mmol/L oligomycin and 80 mmol/L 2-deoxy-d-glucose (2-DG). OCR was assessed under basal conditions and after sequential injection of 1 μmom/L oligomycin, 1 μmol/L fluoro-carbonyl cyanide phenylhydrazone (FCCP) and 2 mmol/L antimycin A and rotenone.

### Glucose assays

(1) Cellular glucose uptake was determined by flow cytometry analysis of uptake of 2-[N-(7-nitrobenz-2-oxa-1, 3-diazo-l-4-yl) amino]-2-deoxy-d-glucose (2-NBDG). Breast cancer cells were incubated with 50 μmol/L of 2-NBDG for 30 min at 37 °C in a humidified atmosphere containing 5% CO_2_/95% air. The fluorescence intensity of 2-NBDG taken up by the cells was analyzed on an EasyCyte Plus Flow Cytometry System (Millipore, Chicago, IL, USA). (2) The glucose concentration in the medium was measured using an Amplex Red Glucose/Glucose oxidase assay kit (Invitrogen). The absorbance of the samples was measured using a Varioskan multimode microplate spectrophotometer (Thermo, MA) and used to calculate the concentration of glucose in the medium.

### Measurement of lactate and ATP levels

Lactate levels in the extracellular medium and the intracellular lactate levels (determined from cell lysates) were measured using a lactate assay kit (Abcam, Cambridge, UK). Data were normalized to the final cell counts. ATP content was determined by collecting the cells and lysing them in ATP lysis buffer, followed by ATP concentration determinations using an ATP assay kit (Beyotime Biotechnology, Shanghai, China) and following the manufacturer's protocol.

### PFKP enzyme activity

Cell lysates were added into to a reaction mixture containing 50 mmol/L Tris–HCl (pH = 8.0), 50 mmom/L potassium fluoride and 2.5 mmol/L EDTA, 100 mmol/L KCl, 5 mmol/l MgCl_2_, 1 mmol/L ATP, 0.2 mmol/lL NADH, 5 mmol/L Na_2_HPO_4_, 0.1 mmol/L AMP, 1 mmol/L NH_4_Cl, 5 mmol/L fructose-6-phosphate, 5 units triose phosphate isomerase, 1 unit aldolase, and 1 unit α-glycerophosphate dehydrogenase. The absorbance at 340 nm was measured at room temperature using a spectrophotometer. PFKP activity was represented as the percent loss of DPNH in the reaction mixture.

### Plasmid construction

WT-PFKP or PFKP mutants (S386D-PFKP, S386A-PFKP, and K10R-PFKP) were generated using the QuikChange Site-Directed Mutagenesis kit (Stratagene, La Jolla, California) according to the manufacturer’s instructions.

### Ubiquitylation assay

The in vitro ubiquitylation assay was conducted by incubating purified WT HA-HRD1 (2 μg) or HA-C291S (a HRD1 ligase-dead mutant) (2 μg) with purified His-PFKP and 100 nmol/L E1, 2 μmol/L His-E2 (Ubc4), 10 μmol/L GST-Ub, and 2 mmol/L ATP in a reaction buffer (50 mmol/L Tris–HCl [pH = 7.5], 2.5 mmol/L MgCl_2_, and 0.5 mmol/L DTT) for 90 min at room temperature.

The in vivo ubiquitylation assay was run by transfecting MDA-MB-231 cells with ubiquitin-HA for 48 h, followed lysis in the denaturing buffer (6 mol/L guanidine-HCl [pH = 8.0], 0.1 mol/L Na_2_HPO_4_/NaH_2_PO_4_, and 10 mmol/L imidazole) containing 5 mmol/L N-ethylmaleimide to prevent de-ubiquitylation. The cell lysates were immunoprecipitated using the appropriate antibodies and the precipitates were washed and subjected to immunoblotting analysis.

### Co-immunoprecipitation (Co-IP) and immunofluorescence (IF) staining

MDA-MB-231 cells were grown to confluence and processed for Co-IP by standard procedures. Briefly, the cells (5 × 10^7^) were lysed in cold lysis buffer, centrifuged, and 10% of the supernatants were used as inputs. The remaining supernatants were immunoprecipitated with protein A/G agarose beads, IgG (negative controls), or antibody for target proteins at 4 °C overnight. After washing, the pellets were suspended in 2 × SDS and subjected to western blotting.

The IF staining assay was performed using standard protocols. The MDA-MB-231 cells were fixed and incubated with primary antibodies (anti-HRD1 or Anti-PFKP) at a dilution of 1:100, fluorescent dye-conjugated secondary antibodies, and 4′,6′-diamidino-2-phenylindole (DAPI). Images were acquired with a laser scanning microscope (Olympus).

### Migration, invasion, cell proliferation, and colony-formation assays

Stable cell lines of MCF-7 and MB231 cells overexpressing HRD1 were constructed and cultured as previously reported [17]. Stable cell lines were treated with 5 mmol/L 2-deoxy-d-glucose (2-DG) for 72 h and then cell proliferation was measured using CCK-8 assays. The migration and invasion assays were conducted on stable cell lines treated with 5 mmol/L 2-DG for 48 h and then placed in serum-free media. The colony-formation assays were performed by seeding stable cell lines in 6-well plates at a density of 200 cells per well and treating them with 5 mmol/L 2-DG for 3 weeks.

#### Western blotting

Cells were washed twice in ice-cold PBS and then solubilized in radioimmunoprecipitation assay (RIPA) lysis buffer (Vazyme, Nanjing, China). Samples containing equal amounts of protein were analyzed by western blotting as previously reported [18].

#### Real-time PCR assay

Total RNA was isolated using TRIzol reagent (Invitrogen) following the manufacturer’s protocol. The mRNA was quantified by real-time PCR using a LightCycler480 II Sequence Detection System (Roche, Basel, Switzerland). The following primers were used to identify PFKP: forward, 5′-CGG AAG TTC CTG GAG CAC CTC TC-3′ and reverse, 5′-AAG TAC ACC TTG GCC CCC ACG TA-3′. GAPDH was used as an internal control: forward, 5′-CCC CTT CAT TGA CCT TCA ACT A-3′ and reverse, 5′-GAG TCC TTC CAC GAT ACC AAA G-3′. All reactions were performed in triplicate, and the relative PFKP mRNA level was calculated using the 2^−ΔΔCT^ method.

#### Animal tumor model

All mouse experiments were approved by the Committee on the Ethics of Animal Experiments of Nanjing Medical University. MDA-MB-231 cells (1 × 10^7^ in 100 µL PBS) stably expressing HRD1 with or without overexpression of PFKP were injected into the flank region of 6- to 8-week-old female BALB/c nude mice. The tumor size was measured every 4 days, beginning one week after the implantation, and the tumor volume was analyzed using the formula V = 0.5 × length × width^2^.

Cells were injected into tail vein of the mice to generate the lung metastasis model. The mice (ten mice per group) were sacrificed 6 weeks after the injection. The size and weight of the lungs were assessed, and visible tumors on the lung surface were counted.

#### Immunohistochemistry (IHC) staining

Tumor tissues used for IHC staining were measured as described previously [17]. The primary antibody against HRD1 (1:200) anti-PFKP (1:200), and secondary antibody (1:1000) were used. Diaminobenzidine (DAB) substrate solution was applied before hematoxylin counterstaining. Normal IgG was used for negative control assays. Images were captured and evaluated with a laser scanning microscope (Olympus).

#### Statistical analysis

Statistical analyses were performed using SPSS 19.0 statistical analysis software. Data were expressed as the mean ± SD. Analysis of variance (ANOVA) was used to determine the statistical differences among the groups. A multivariate analysis of the independent prognostic factors was conducted using the Cox proportional hazards model. A *P* value < 0.05 was considered statistically significant.

## Results

### Upregulation of HRD1 led to an obvious decrease in aerobic glycolysis in breast cancer cells

**Fig. 1:**
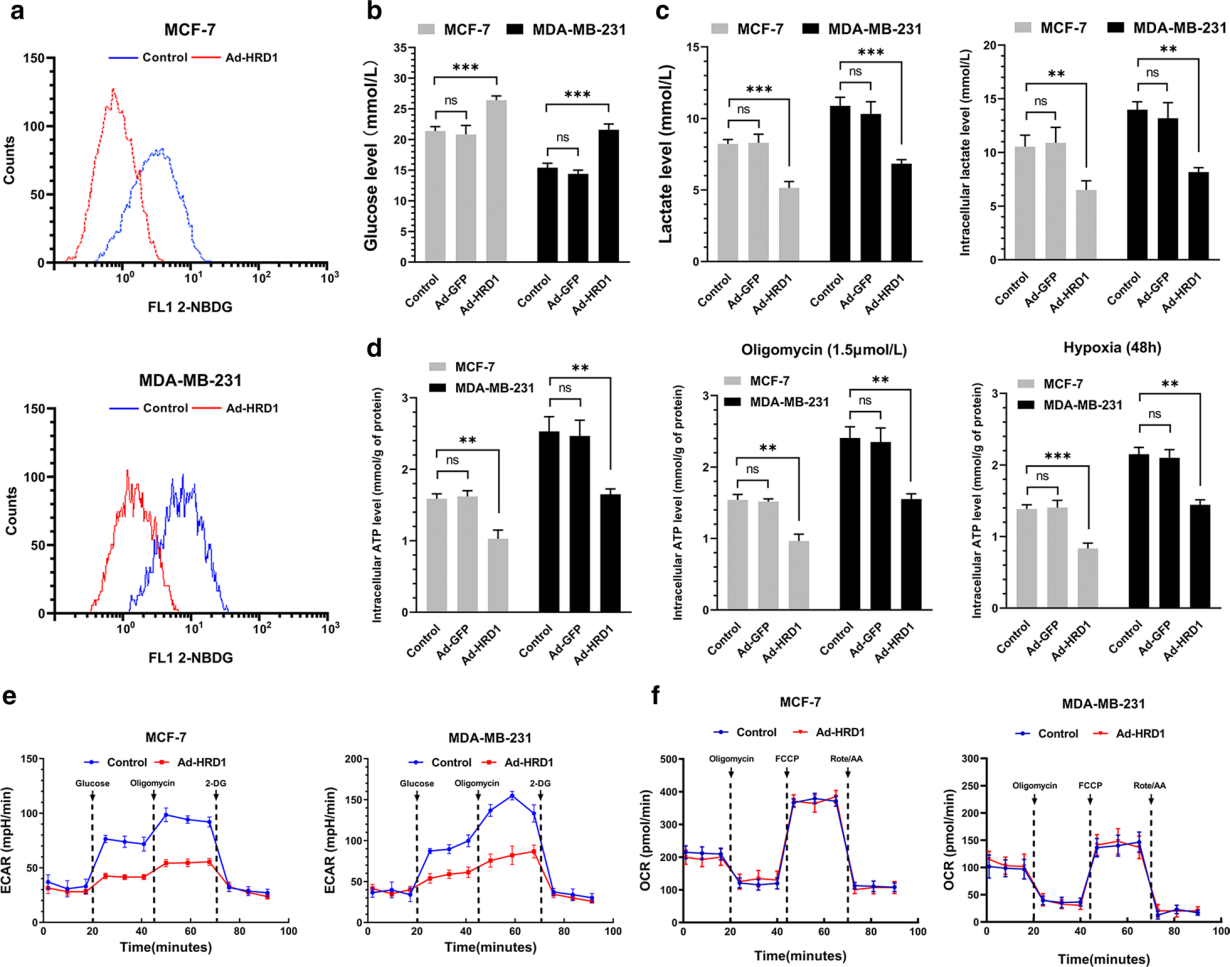
Upregulation of HRD1 led to an obvious decrease in robic glycolysis in breast cancer cells. MCF-7 and MDA-MB-231 cells were infected with Ad-GFP or Ad-HRD1 for 48 h. At the end of infection, **a** glucose uptake was determined by measuring uptake of 2-NBDG using flow cytometry. **b** Glucose concentration in the medium was measured using the AmplexRed glucose/glucose oxidase assay kit. **c** Lactate levels in the extracellular medium and the intracellular lactate levels in the cell lysates were measured using a lactate assay kit. **d** ATP concentration was measured using an ATP assay kit. **e** Extracellular acidification rate (ECAR) and **f** oxygen consumption rate (OCR) were measured using a Seahorse XF96 Flux Analyzer. Each point represents the mean ± SD of triplicate determinations

The role of HRD1 in breast cancer metabolism was examined by upregulating, HRD1 expression in MCF7 and MDA-MB-231 cells using lentivirus that over-expressed HRD1, as described previously [17]. The resulting breast cancer cells that overexpressed HRD1 exhibited a significant decrease in glucose uptake, while the glucose level was significantly higher in the medium surrounding these cells (Fig. 1a, b). Consistently, HRD1 overexpression reduced lactate production and cellular ATP levels (Fig. 1c, d). The potential regulatory role of HRD1 in breast cancer glucose metabolism was further explored by ECAR and OCR assays to characterize the metabolic alterations in glycolysis due to HRD1 overexpression. HRD1 overexpression reduced ECAR (Fig. 1e), but had no effect on OCR (Fig. 1f). These data clearly supported a role for HRD1 in inhibiting glycolysis but not the tricarboxylic acid (TCA) cycle.

### HRD1 elicited an anti-Warburg effect that inhibited growth, migration, and invasion of breast cancer cells

**Fig. 2:**
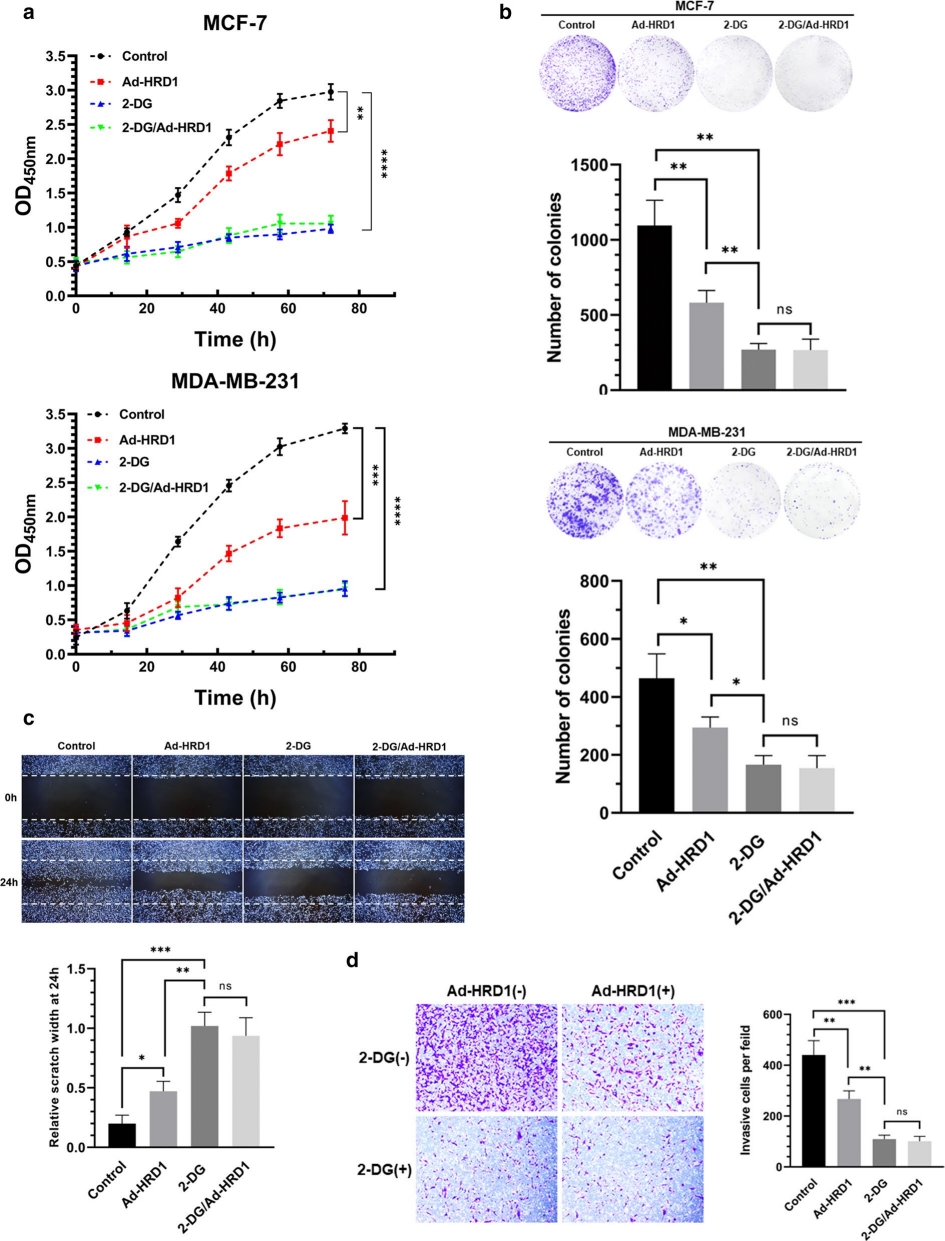
HRD1 elicited an anti-Warburg effect that inhibited the growth, migration, and invasion of breast cancer cells. **a** MCF-7 and MDA-MB-231 cells stably expressing HRD1 were treated with 2-deoxyglucose (2-DG; 5 mmol/L) for 72 h, and then cell proliferation was measured using CCK-8 assays. **b** MCF-7 and MDA-MB-231 cells stably expressing HRD1 were treated with 2-DG (5 mmol/L) for 3 weeks, and then colony-formation assay were performed. **c**, **d** MCF-7 and MDA-MB-231 cells stably expressing HRD1 were treated with 2-DG (5 mmol/L) for 48 h, and then the migration and invasion assays were performed

Our previous study demonstrated that upregulation of HRD1 inhibited the growth and proliferation of breast cancer cells [17]. The importance of the Warburg effect in HRD1-mediated anti-oncogenesis was investigated by treating breast cancer cells with 2-DG to inhibit glycolysis. CCK-8 assays confirmed that 2-DG inhibited the growth of MCF-7 and MDA-MB-231 cells (Fig. 2a), which was similar to previous study [20]. Notably, 2-DG also abolished the suppressive effect of HRD1 overexpression on the growth of breast cancer cells (Fig. 2a) as well as the inhibitory effect of HRD1 overexpression on colony-formation by MCF-7 and MDA-MB-231 cells (Fig. 2b). The suppression of migration and invasion induced by HRD1 overexpression was also abolished by 2-DG (Fig. 2c, d).

### HRD1 decreased the protein stability of PFKP

**Fig. 3:**
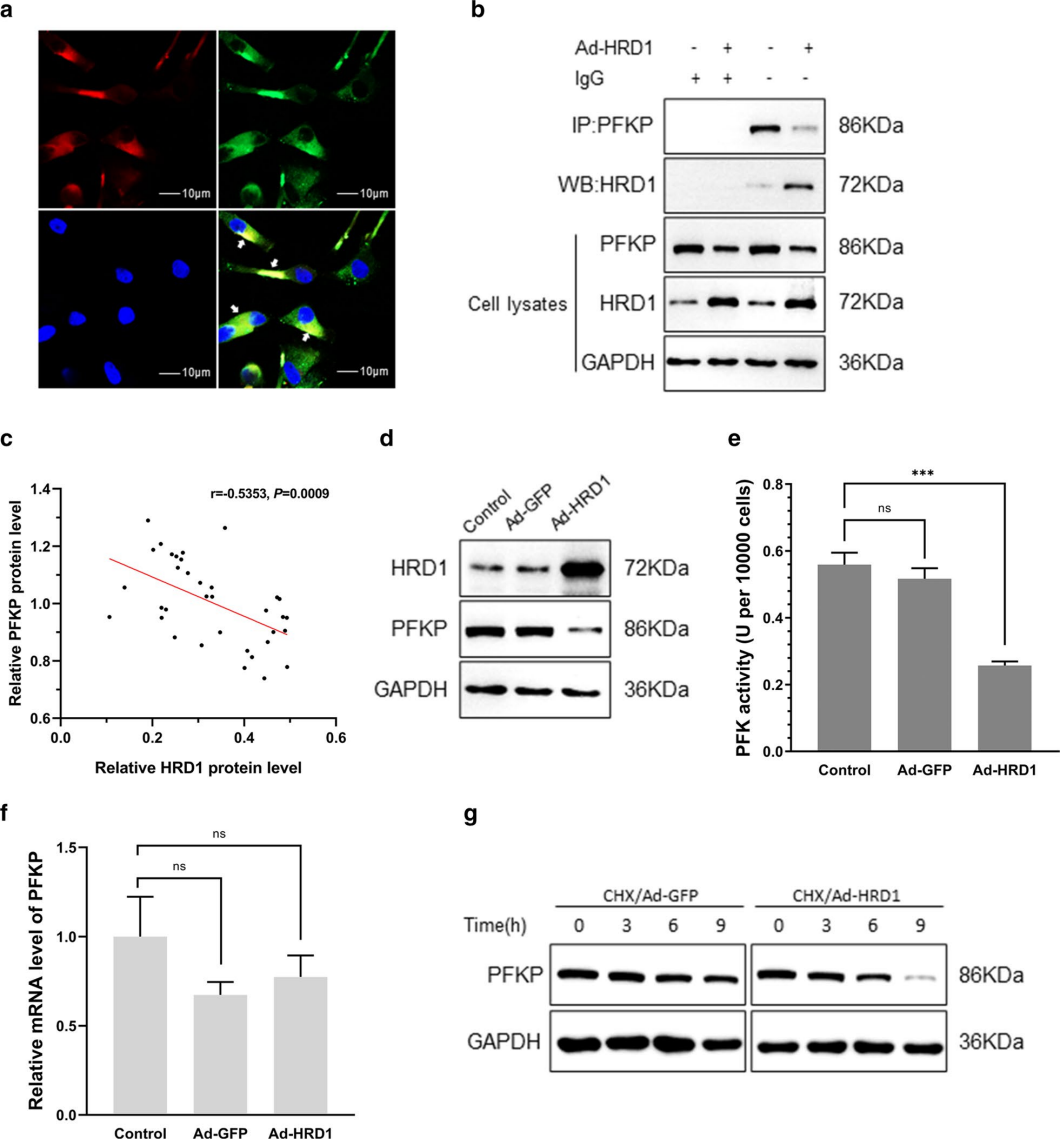
HRD1 decreased protein stability of PFKP. **a**, **b** MDA-MB-231 cells were infected with Ad-GFP or Ad-HRD1 for 48 h. Immunofluorescence staining was performed for PFKP (red), HRD1 (green), and DAPI (blue). Scale bars = 10 μm. Arrows indicate the area where PFKP and HRD1 co-localized in cytoplasm. Co-immunoprecipitation assays were performed to measure the interaction between PFKP and HRD1. **c** Western blotting was performed to measure the level of HRD1 and PFKP proteins in each breast cancer tissue (35 cases). The relative amounts of HRD1 and PFKP protein in each tissue are expressed by the grey density of each protein per grey density of GAPDH protein. **d**–**f** The protein level, enzyme activity and mRNA level of PFKP in MDA-MB-231 cells infected with Ad-GFP or Ad-HRD1 for 48 h were measured by western blotting, PFKP enzyme activity assay and real-time PCR assays, respectively. **g** MDA-MB-231 cells were infected with Ad-GFP or Ad-HRD1 for 48 h. After co-culture with cycloheximide (CHX, 50 mmol/L) for 0, 3, 6, or 9 h, western blotting was performed, and the relative PFKP expression was calculated. Data are presented as mean ± SD and represent three separate experiments

The molecular mechanism of HRD1 inhibition of growth of breast cancer cells was explored by mass spectrometry to investigate the proteins that bind to HRD1 in MDA-MB-231 cells. These experiments identified PFKP as one of the candidate substrates of HRD1 in breast cancer cells (Supplementary Table S1). Co-IP and IF staining assays confirmed a direct interaction between HRD1 and PFKP (Fig. 3a, b), and the interaction was intensified by overexpression of HRD1 (Fig. 3b). The Hrd1 and PFKP protein levels were negatively correlated in breast cancer tissues (*P* < 0.001 by Spearman’s correlation test; Fig. 3c).

The expression and activity of PFKP is mainly regulated through post-translational modifications [11]. HRD1 overexpression reduced the PFKP protein level and PFKP activity in MDA-MB-231 cells but had no effect on the level of PFKP mRNA (Fig. 3d–f). Treatment of MDA-MB-231 cells with cycloheximide (CHX) increased the rate of PFKP degradation in HRD1-overexpressing cells (Fig. 3g). However, overexpression of a HRD1 ligase-dead mutant (C291S) had no effect on PFKP expression, activity or protein stability in MB231 cells (Additional file 1: Fig. 1A–C). These data indicated that HRD1-mediated PFKP degradation was dependent on HRD1 enzyme activity.

### HRD1 acted as an E3 ubiquitin ligase for PFKP degradation

**Fig. 4:**
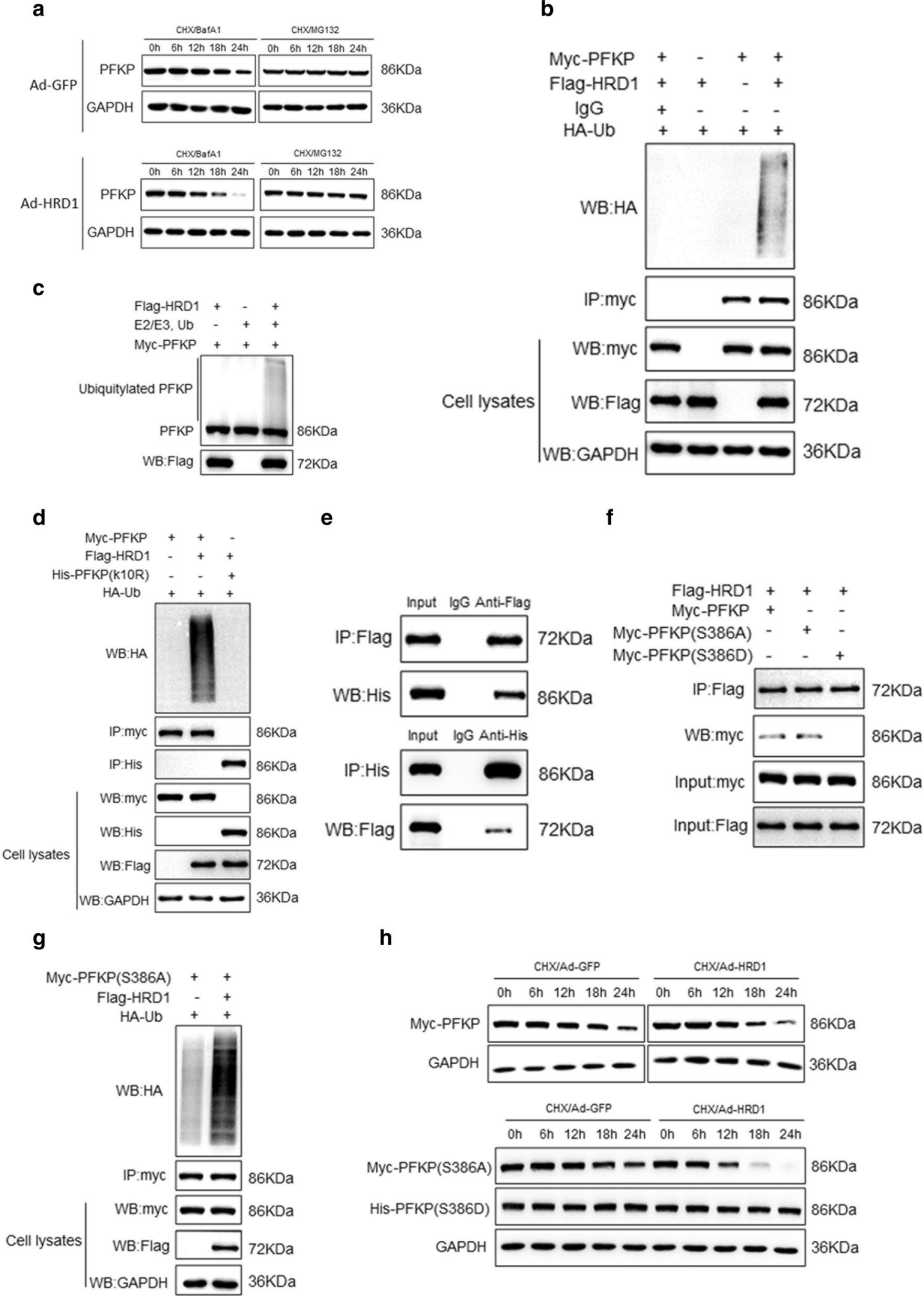
HRD1 acted as an E3 ubiquitin ligase for PFKP degradation. **a** MDA-MB-231 cells were infected with Ad-GFP or Ad-HRD1 for 48 h. After treatment with a proteasome inhibitor MG132 (10 μmol/L) or an autophagy inhibitor bafilomycin A1 (BafA1, 10 nmol/l) for 6 h, the cells were lysed, and western blotting was performed. **b** MDA-MB-231 cells were transfected with Ub-HA and myc-HRD1 or vector control for 48 h. Cell lysates were immunoprecipitated with anti-HA antibody and then detected by western blotting with anti-PFKP and anti-myc to measure the level of endogenous PFKP ubiquitination. **c** Ubiquitination of bacterially expressed His-PFKP by purified HRD1 was determined in vitro. **d** PFKP-depleted MDA-MB-231 cells were co-transfected with HA-Ub, Myc-tagged HRD1 and WT Flag-PFKP or Flag-PFKP K10R mutants. Cell lysates were IP with anti-HA antibody and then detected by western blotting with anti-Flag and anti-Myc. **e** PFKP-depleted MDA-MB-231 cells were co-transfected with Myc-tagged HRD1 and WT Flag-PFKP or Flag-PFKP K10R mutant. Cell lysates were IP with anti-Myc antibody and then detected by Western blot with anti-Flag. **f** PFKP-depleted MDA-MB-231 cells with were co-transfected with Myc-tagged HRD1 and WT Flag-PFKP or Flag-PFKP S386A. Cell lysates were IP with anti-Myc antibody and then detected by western blotting with anti-Flag. **g** PFKP-depleted MDA-MB-231 cells were co-transfected with HA-Ub, Myc-tagged HRD1 and WT Flag-PFKP or Flag-PFKP S386A. Cell lysates were IP with anti-HA antibody and then detected by western blotting with anti-Flag and anti-Myc. **h** PFKP-depleted MDA-MB-231 cells with were co-transfected with Myc-tagged HRD1 and WT Flag-PFKP or Flag-PFKP S386A or Flag-PFKP S386D. Cells were treated with CHX (50 mmol/l) for the indicated periods of time. The quantification of Flag levels relative to GAPDH levels is shown. Data represent the means ± SD of three independent experiments

The molecular mechanism underlying the HRD1-associated decrease in PFKP protein stability was further evaluated using a proteasome inhibitor (MG132) and an autophagy inhibitor (bafilomycin A1, BafA1). Treatment with MG132, but not with BafA1, dramatically blocked the PFKP degradation induced by HRD1 overexpression (Fig. 4A), indicating that PFKP degradation mainly occurred through the proteasomal pathway. In vitro and in vivo ubiquitination assays confirmed that PFKP was a direct substrate of HRD1 (Fig. 4b, c).

The relationship between PFKP and HRD1 was further examined using the PFKP K10R mutant (K10R-PFKP). K10 is a ubiquitylation residue of PFKP [13], and the PFKP K10R mutant was resistant to HRD1-mediated ubiquitylation when compared with the wild-type PFPK (Fig. 4d). This finding supported K10 as the site for HRD1-induced PFKP ubiquitination, and further study showed that K10R did not affect the PFKP/HRD1 interaction (Fig. 4e). Therefore, the resistance to HRD1-mediated ubiquitination ubiquitination was not due to effects on the interaction between HRD1 and PFKP by K10R.

Phosphorylation of PFKP at Ser386 also blocks its ubiquitylation and degradation. We investigate the importance of this Ser phosphorylation involved in HRD1-mediated PFKP degradation, by mutating this Ser to Asp (S386D) to mimic a constitutively phosphorylated form and by mutating the Ser to Ala (S386A) to prevent phosphorylation. HRD1 could bind to WT-PFKP and S386A-PFKP, but not to S386D-PFKP (Fig. 4f). In addition, the S386A-PFKP had a higher ubiquitylation level in MDA-MB-231 cells expressing HRD1 than in MDA-MB-231 cells transfected with a control vector. By contrast, S386D-PFKP was hardly ubiquitinated in either cell type (Fig. 4g). Similarly, overexpression of HRD1 promoted S386A-PFKP degradation but had no effect on S386D-PFKP stability (Fig. 4h).

### HRD1 inhibited aerobic glycolysis, growth, migration, and invasion of breast cancer cells via PFKP downregulation

**Fig. 5:**
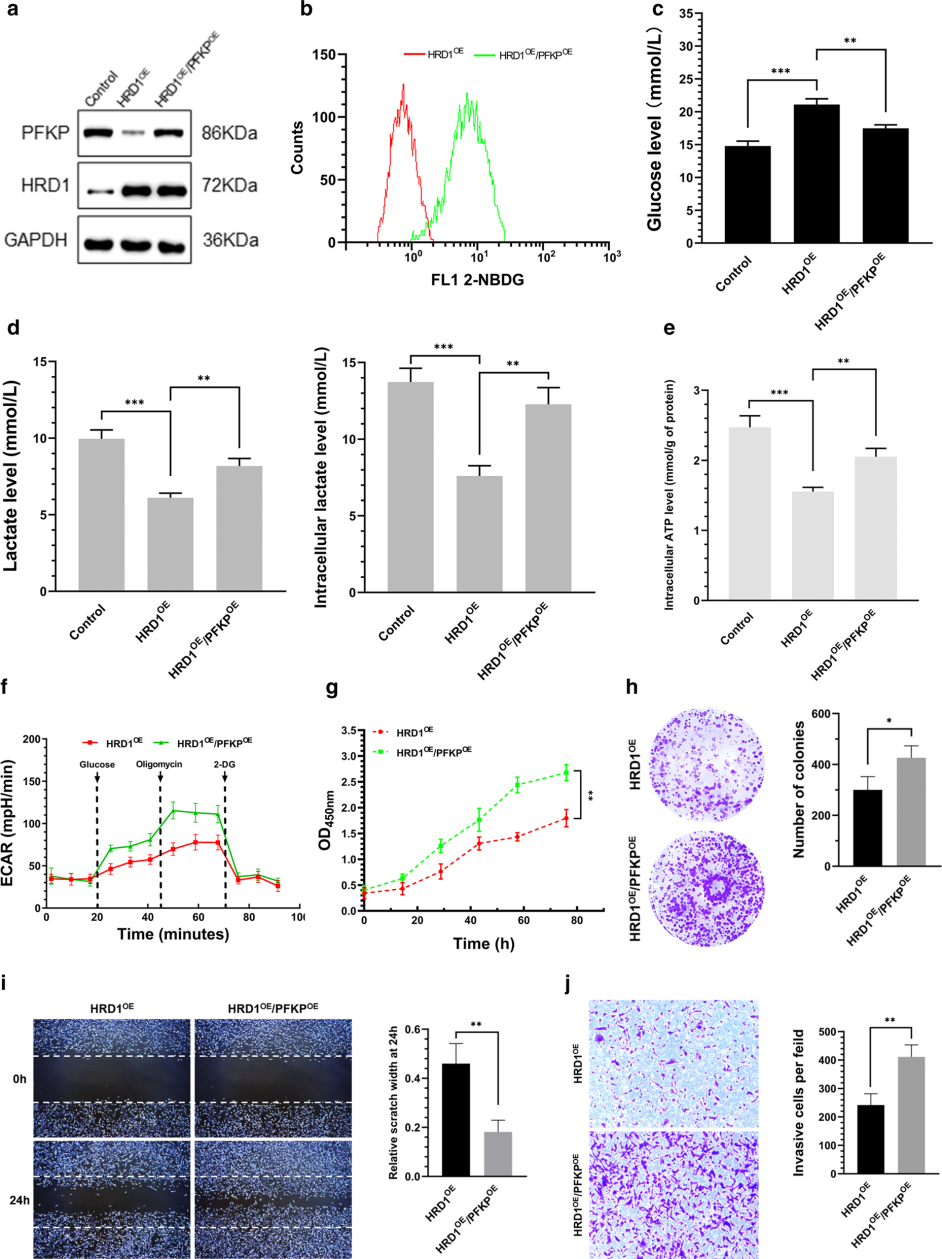
HRD1 inhibited aerobic glycolysis, growth, migration, and invasion of breast cancer cells via PFKP downregulation. MDA-MB-231 cells stably expressing HRD1 were infected with a lentivirus for PFKP for 48 h. **a** Western blotting was performed. **b** Glucose uptake was determined by measuring uptake of 2-NBDG using flow cytometry. **c** Glucose concentration in the medium was measured using the Amplex Red glucose/glucose oxidase assay kit. **d** Lactate levels in the extracellular medium and the intracellular lactate levels in the cell lysates were measured using the lactate assay kit. **e** ATP concentration was measured using an ATP assay kit. **f** Extracellular acidification rate (ECAR) was measured using Seahorse XF96 Flux Analyzer. **g** MDA-MB-231 cells stably expressing HRD1 were infected with a lentivirus for PFKP for 72 h, and then cell proliferation was measured using CCK-8 assays. **h** MDA-MB-231 cells stably expressing HRD1 were infected with a lentivirus for PFKP for 3 weeks, and then colony-formation assay were performed. **i**, **j** MDA-MB-231 cells stably expressing HRD1 were infected with a lentivirus for PFKP for 48 h, and then the migration and invasion assays were performed

The role of PFKP in HRD1-mediated breast cancer inhibition was investigated by overexpressing PFKP in MDA-MB-231 cells stably expressing exogenous HRD1 (Fig. 5a). PFKP was simultaneously upregulated in the HRD1-overexpressing cells and this completely reversed, the decrease in glucose uptake, lactate production, cellular ATP levels, and ECAR caused by overexpression of HRD1 (Fig. 5b–f). The forced expression of PFKP also restored the growth, migration, and invasion that had been reduced by HRD1 overexpression in MDA-MB-231 cells (Fig. 5g–j). Similar results were also observed in MCF-7 cells (Additional file 2: Fig. 2). Therefore, HRD1 appeared to inhibit aerobic glycolysis, growth, migration, and invasion of breast cancer cells by downregulating PFKP.

### HRD1 inhibited breast cancer growth and metastasis in vivo through a PFKP-dependent way

**Fig. 6:**
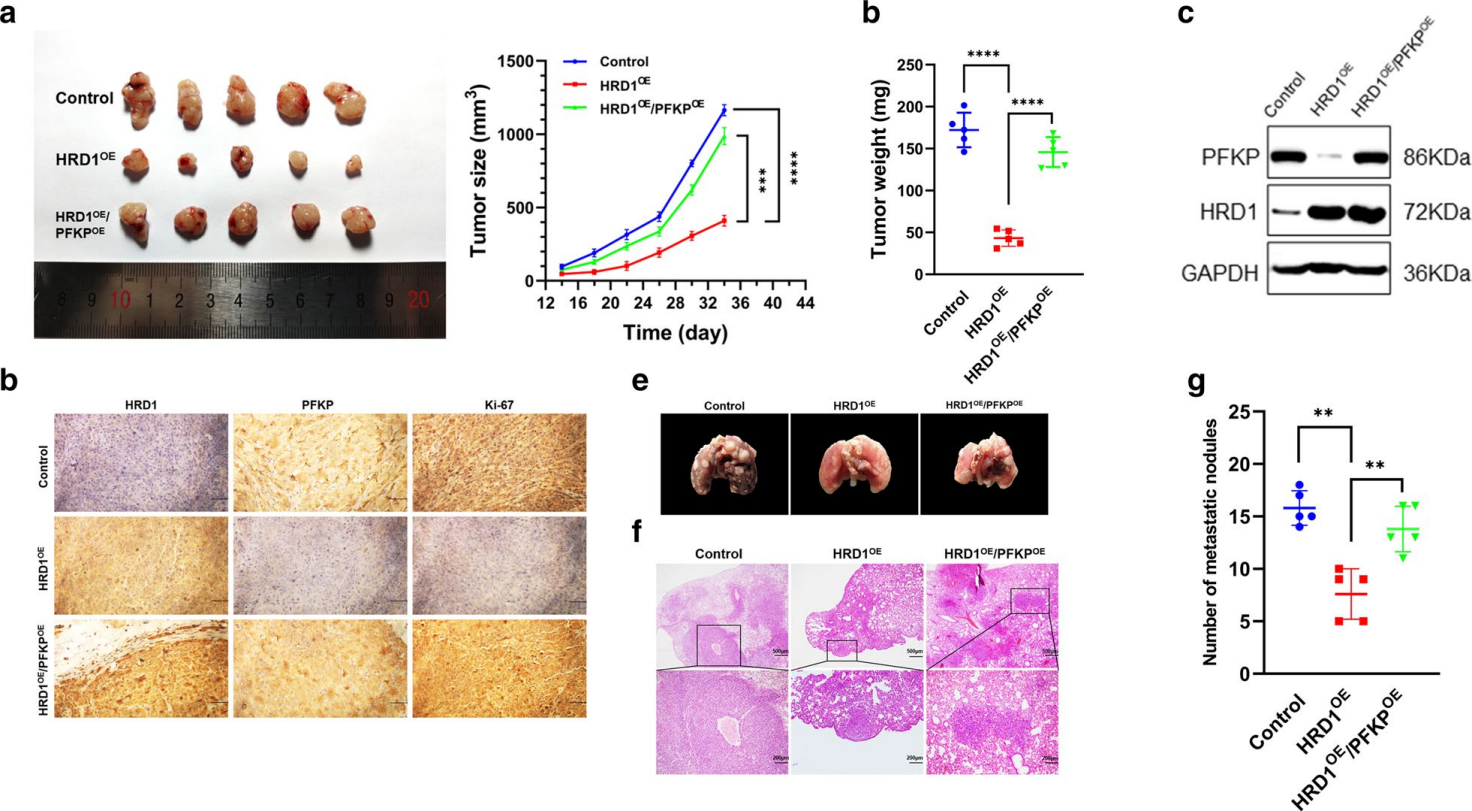
PFKP was responsible for HRD1-mediated inhibition of breast cancer growth and metastasis in vivo. MDA-MB-231 cells stably expressing HRD1 were infected with a lentivirus of PFKP and injected into the right flank and left flank of nude mice. **a** Tumor sizes were monitored twice per week and **b** tumor weights were measured. The expression of PFKP in tumor was measured using western blotting (**c**) and immunohistochemistry (**d**). MDA-MB-231 cells stably expressing HRD1 infected with or without a lentivirus for PFKP were inoculated into nude mice via the tail vein. After 5 weeks, representative images of metastases in mice lung (**e**), H&E staining (**f**) and the numbers of tumor nodules on the lung surfaces (**g**) in each experimental group were examined

The role of PFKP in breast cancer tumorigenesis was investigated by intracranial injection of MDA-MB-231 cells stably expressing HRD1, with or without overexpression of PFKP, into athymic nude mice. HRD1 overexpression inhibited the tumor growth, whereas ectopic expression of PFKP reversed this inhibition (Fig. 6a, b). Western blotting and immunohistochemistry analyses revealed that overexpression of PFKP could restore PFKP levels in the tumor tissues expressing HRD1 (Fig. 6c, d). Consistently, inoculation of MDA-MB-231 cells stably expressing HRD1 decreased the number of metastatic nodules, whereas this effect was negated by overexpressing PFKP (Fig. 6e–g).

## Discussion

**Fig. 7:**
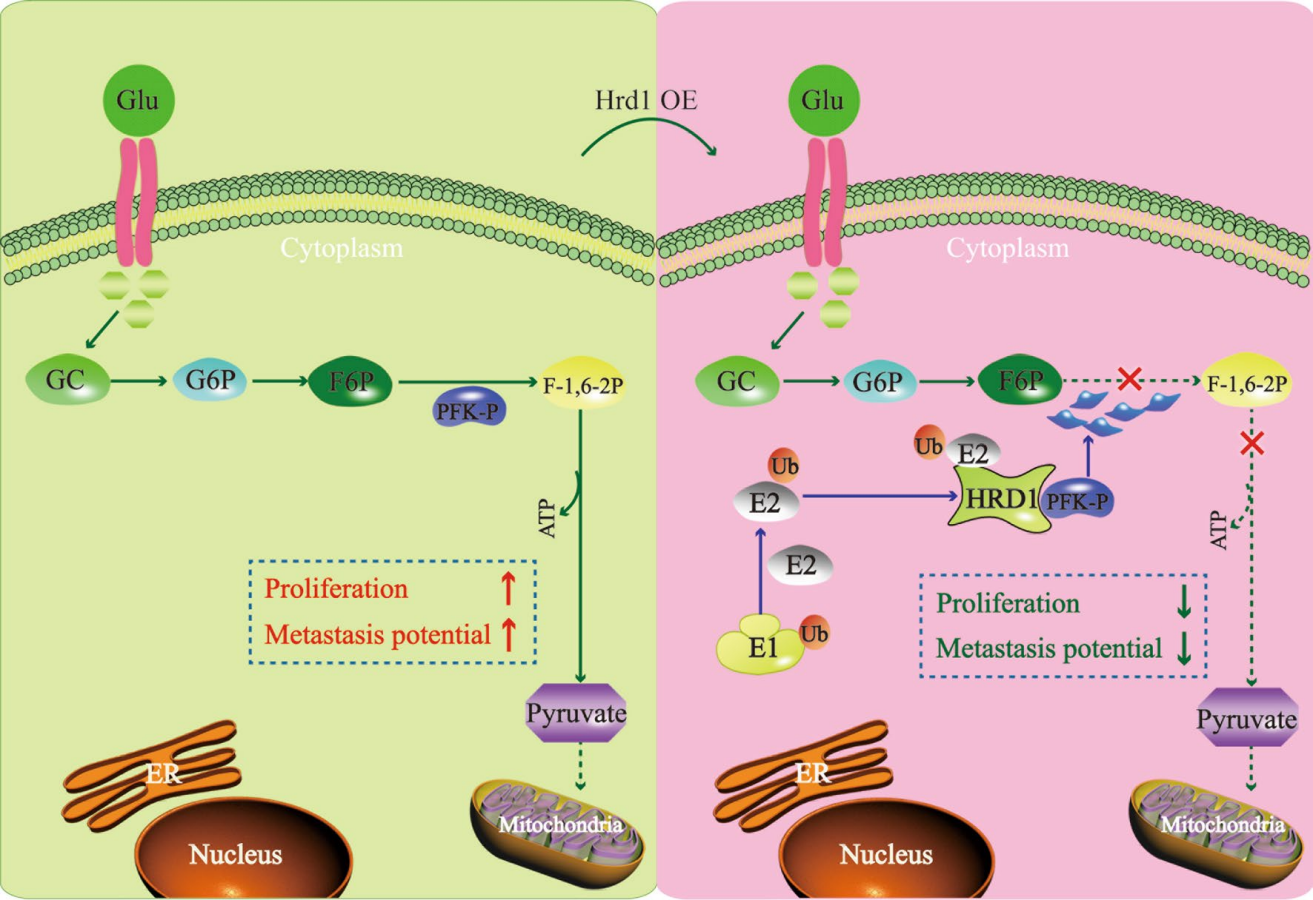
A model to depict the role of HRD1 in an anti-Warburg effect in breast cancer

Growing evidence indicates that aerobic glycolysis is the driving force of tumorigenesis in breast cancers [21, 22]. Here, we identified that elevated expression of HRD1 elicited an anti-Warburg effect, and inhibited breast cancer growth and metastasis both in vitro and in vivo. Mechanistically, HRD1 functions as an E3 ubiquitin ligase that catalyzed PFKP ubiquitination and promoted PFKP degradation. A model to depict the role of HRD1 in an anti-Warburg effect in breast cancer is presented (Fig. 7).

In recent years, the rapid development of metabolomics has revealed that a metabolic disorder plays an important role in the occurrence, development, and consequence of cancer [23]. Meanwhile, ‘anti-Warburg’ action creates opportunities to predict and prevent cancer growth based on individualized patient profiles, and the application of targeted ‘anti-Warburg’ measures enhances the therapeutic efficacy in breast cancer management [24–26]. However, the regulatory mechanism of the Warburg Effect in breast cancer is still not fully understood. In the present study, we found that E3 ligase HRD1 could inhibit aerobic glycolysis in breast cancer cells, providing new evidence for a role as a regulator of tumor metabolism. Our previous studies had revealed that HRD1 had a tumor suppressive effect that involved suppression of breast cancer cell proliferation, migration and invasion [17–19]. Aerobic glycolysis is well known to be critical in driving breast cancer tumorigenesis and maintaining cancer cell proliferation [27, 28]. Therefore, the anti-Warburg effect of HRD1 in breast cancer cells could contribute to its suppression of tumor development and progression. This possibility was supported by the finding that 2-DG, a nonmetabolisable glucose competitor, abolished the anti-tumor activity of HRD1 (Fig. 2).

As an E3 ligase, HRD1 plays a biological role that is closely related to its substrates. Here, our mass spectrometry analyses identified an interaction between HRD1 and PFKP, the rate-limiting enzyme in glycolysis, in breast cancer cells. The clinical significance of HRD-mediated degradation and decreased stability of PFKP was also indicated by the negative correlation between HRD and PFKP expression in human breast cancer tissues. The ubiquitination assays further confirmed that PFKP was a direct substrate of HRD1 and that HRD1 ubiquitinated PFKP at lysine 10. More importantly, restoration of PFKP expression abolished the tumor suppressive effects of HRD1 both in vitro and in vivo.

PFKP is overexpressed in breast cancer and promotes the Warburg effect [6, 12], and PFKP expression and activity are mainly regulated by post-translational modifications [11]. Phosphorylation of PFKP at S386 is known to increase stability and pyruvate kinase activity [13], and our PFKP mutation plasmid with this Ser site mutated Asp (S386D-PFKP) to mimic constitutive phosphorylation prevented HRD1-mediated the ubiquitylation and degradation. However, the nonphosophorylatable PFKP mutant (S386A) enhanced the binding of HRD1 to PFKP and had a higher ubiquitylation level than the WT-PFKP. This observation suggested that the non-phosphorylated state at S386 was indispensable for maintaining the PFKP ubiquitination induced by HRD1.

PFKP is phosphorylates by AKT at S386, which blocks the ubiquitylation and degradation of PFKP [13]. AKT activation could trigger the Warburg effect in cancer cells and PI3K-Akt-mTOR pathway is a key intracellular signaling pathway in regulating metabolic checkpoints [29–31]. Our previous study confirmed that HRD1 played inhibitory effect on AKT activation in breast cancer [17]. Here, it has been clearly suggested the novel role of HRD1 in cancer metabolism. In addition, we found that HRD1 could induce cell arrest in the G2/M phase (data not shown). All the findings suggest that HRD1 could be involved in metabolic check points through AKT/PI3K/mTOR. We will explore whether HRD1 agonist can regulate the expression of cell cycle check point proteins and act as a therapeutic target for treatment of breast cancer in future.

## Conclusion

In summary, this study revealed a novel role for HRD1 in tumor metabolism. HRD1 inhibited glycolysis and tumor growth and metastasis through direct inhibition of PFKP expression in breast cancer cells. To our knowledge, this is the first report that HRD1 down-regulated PFKP by promoting PFKP degradation in human breast cancer cells, thereby eliciting an anti-Warburg effect and inhibiting breast cancer progression.

## Supplementary information

**Additional file 1: Figure 1.** HRD1 ligase-dead mutant (C291S) had no effect on PFKP expression, activity, and protein stability. (A, B C) The protein level, enzyme activity and mRNA level of PFKP in MDA-MB-231 cells infected with Ad-GFP or Ad-HRD1 mutant (C291S) for 48 h were measured by western blotting, PFKP enzyme activity assays and real-time PCR assays, respectively.

**Additional file 2: Figure 2.** HRD1 inhibited aerobic glycolysis and growth of breast cancer cells via PFKP downregulation. MCF-7 cells stably expressing HRD1 were infected with a lentivirus for PFKP for 48 h. (A) Western blot analysis was performed. (B) Glucose uptake was determined by measuring uptake of 2-NBDG using flow cytometry. (C) Glucose concentration in the medium was measured using the Amplex Red glucose/glucose oxidase assay kit. (D) Lactate levels in the extracellular medium and the intracellular lactate levels in the cell lysates were measured using the lactate assay kit. (E) ATP concentration was measured using an ATP assay kit. (F) Extracellular acidification rate (ECAR) was measured using a Seahorse XF96 Flux Analyzer. (G) MCF-7 cells stably expressing HRD1 were infected with a lentivirus of PFKP for 72 h, and then cell proliferation was measured using CCK-8 assays.

## Data Availability

All data generated or analyzed during this study are included in this article.

## Abbreviations

HRD1: HMG-CoA reductase degradation 1
PFKP: Phosphofructokinase
ECAR: Extracellular acidification rate
OCR: Oxygen consumption rate
2-DG: 2-Deoxy-d-glucose
FCCP: Fluoro-carbonyl cyanide phenylhydrazone
2-NBDG: 2-[N-(7-nitrobenz-2-oxa-1, 3-diazo-l-4-yl) amino]-2-deoxy-d-glucose
Co-IP: Co-immunoprecipitation
IF: Immunofluorescence
DAPI: 4′,6′-Diamidino-2-phenylindole
DAB: Diaminobenzidine
ANOVA: Analysis of variance
CHX: Cycloheximide
