# Correction to: Anti-Warburg effect by targeting HRD1-PFKP pathway may inhibit breast cancer progression

**DOI:** 10.1186/s12964-021-00803-1

**Published:** 2021-11-15

**Authors:** Ya Fan, Jia Wang, Yuemei Xu, Yipin Wang, Tao Song, Xiubin Liang, Feng Jin, Dongming Su

**Affiliations:** 1 Department of Pathology, Nanjing Medical University, Nanjing, Jiangsu People’s Republic of China; 2 Department of Breast Surgery, Institute of Breast Disease, The Second Hospital of Dalian Medical University, Dalian, Liaoning People’s Republic of China; 3 Department of Pathology, Nanjing Drum Tower Hospital, The Affiliated Hospital of Nanjing University Medical School, Nanjing, Jiangsu People’s Republic of China; 4 Center of Pathology and Clinical Laboratory, Sir Run Run Hospital of Nanjing Medical University, Nanjing, Jiangsu People’s Republic of China; 5 Department of Pathophysiology, Nanjing Medical University, Nanjing, Jiangsu People’s Republic of China; 6 Department of Breast Surgery, The First Affiliated Hospital of China Medical University, Shenyang, Liaoning Province People’s Republic of China

## Correction to: Cell Commun Signal (2021) 19:18 10.1186/s12964-020-00679-7

Following publication of the original article [1], the authors identified the following error. Due to the improper labelling of cell scratch images during the collection of experimental data, the upper and lower right images of scratch assay in Fig. 5i (i.e. the 0 h and 24 h scratch of cells overexpressing HRD1 and PFKP) were misused, though not affecting the results and scientific conclusions of the original article. The authors apologize for the mistakes and have corrected the error by replacing them with the proper cell scratch images. The updated Fig. 5 is shown as below.

**Fig. 5:**
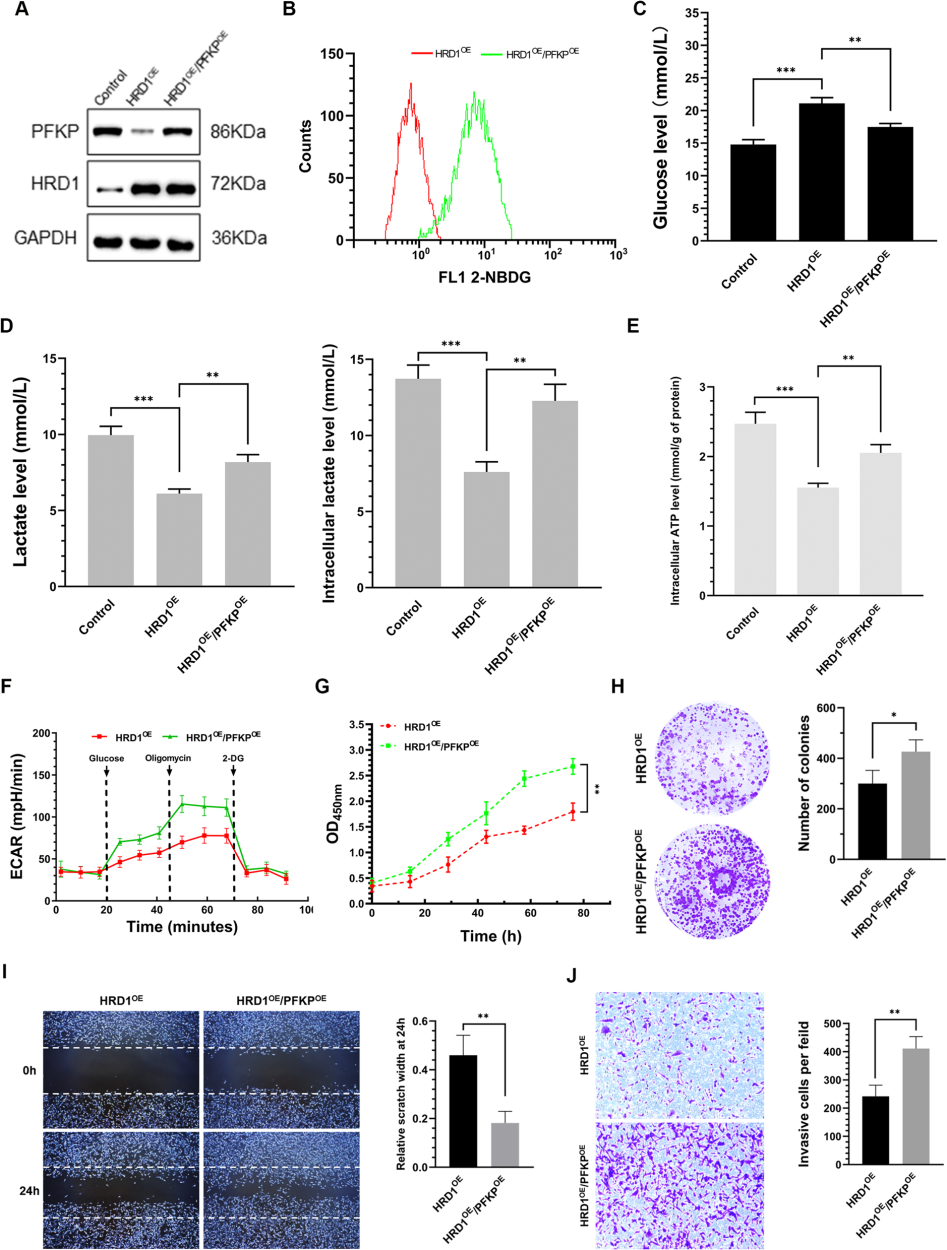
HRD1 inhibited aerobic glycolysis, growth, migration, and invasion of breast cancer cells via PFKP downregulation. MDA-MB-231 cells stably expressing HRD1 were infected with a lentivirus for PFKP for 48 h. **a** Western blotting was performed. **b** Glucose uptake was determined by measuring uptake of 2-NBDG using flow cytometry. **c** Glucose concentration in the medium was measured using the Amplex Red glucose/glucose oxidase assay kit. **d** Lactate levels in the extracellular medium and the intracellular lactate levels in the cell lysates were measured using the lactate assay kit. **e** ATP concentration was measured using an ATP assay kit. **f** Extracellular acidification rate (ECAR) was measured using Seahorse XF96 Flux Analyzer. **g** MDA-MB-231 cells stably expressing HRD1 were infected with a lentivirus for PFKP for 72 h, and then cell proliferation was measured using CCK-8 assays. **h** MDA-MB-231 cells stably expressing HRD1 were infected with a lentivirus for PFKP for 3 weeks, and then colony-formation assay were performed. **i, j** MDA-MB-231 cells stably expressing HRD1 were infected with a lentivirus for PFKP for 48 h, and then the migration and invasion assays were performed
