# Supplementary figures and images for: Anti-Warburg effect by targeting HRD1-PFKP pathway may inhibit breast cancer progression
Source: Cell Commun Signal. 2021 Feb 15;19:18. doi: 10.1186/s12964-020-00679-7 (PMC7883444; doi:10.1186/s12964-020-00679-7)

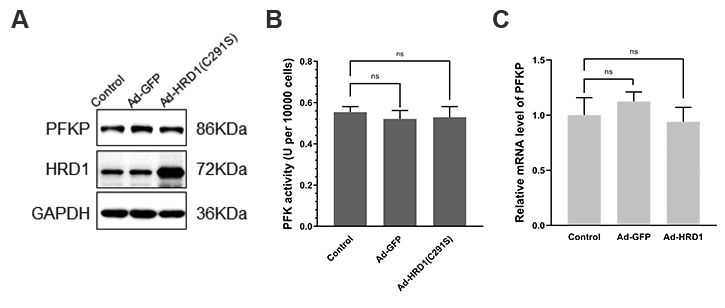

Supplement: Supplementary file 2 — Additional file 1: Figure 1. HRD1 ligase-dead mutant (C291S) had no effect on PFKP expression, activity, and protein stability. (A, B C) The protein level, enzyme activity and mRNA level of PFKP in MDA-MB-231 cells infected with Ad-GFP or Ad-HRD1 mutant (C291S) for 48 h were measured by western blotting, PFKP enzyme activity assays and real-time PCR assays, respectively. [file 12964_2020_679_MOESM2_ESM.tif]

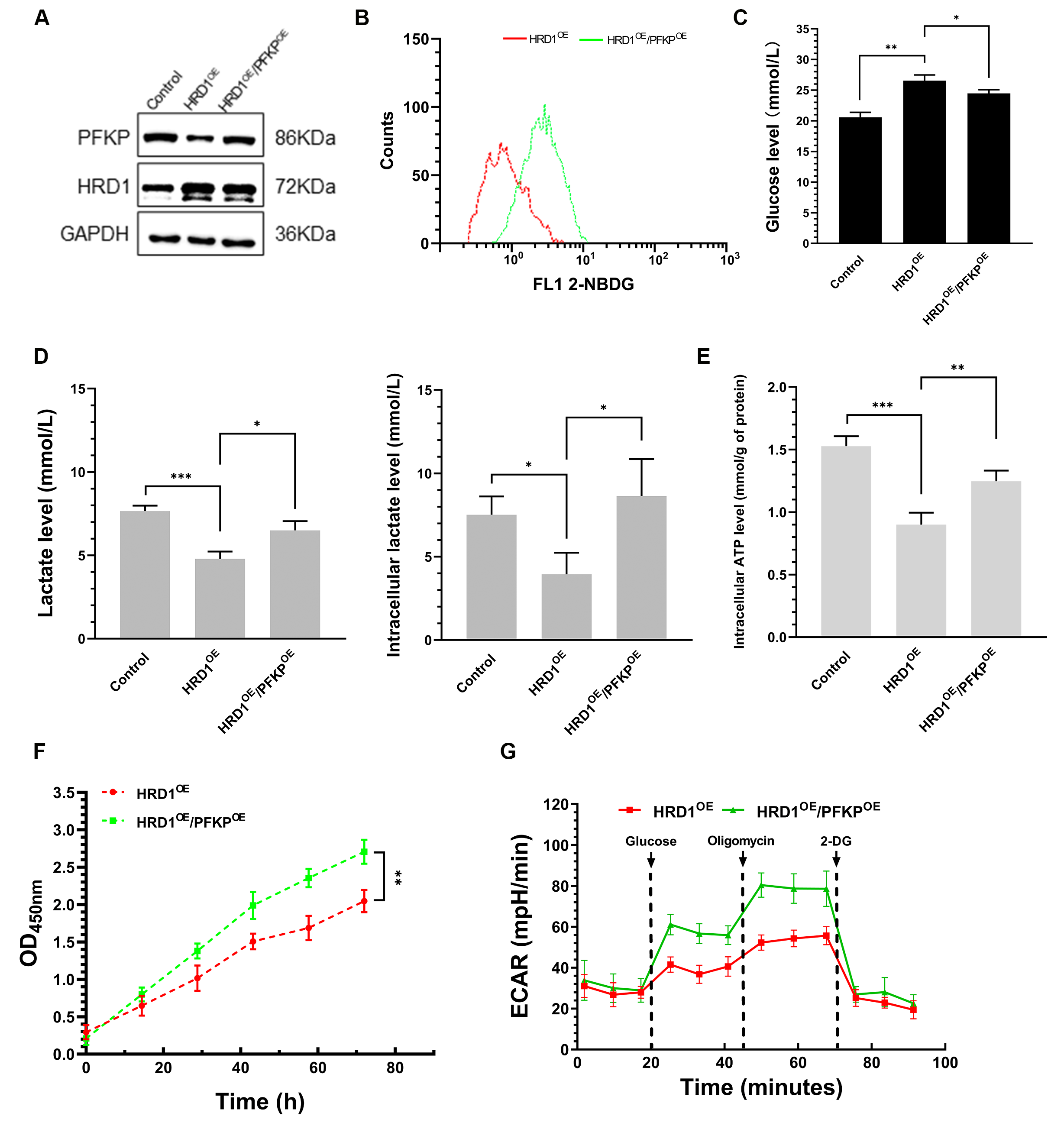

Supplement: Supplementary file 3 — Additional file 2: Figure 2. HRD1 inhibited aerobic glycolysis and growth of breast cancer cells via PFKP downregulation. MCF-7 cells stably expressing HRD1 were infected with a lentivirus for PFKP for 48 h. (A) Western blot analysis was performed. (B) Glucose uptake was determined by measuring uptake of 2-NBDG using flow cytometry. (C) Glucose concentration in the medium was measured using the Amplex Red glucose/glucose oxidase assay kit. (D) Lactate levels in the extracellular medium and the intracellular lactate levels in the cell lysates were measured using the lactate assay kit. (E) ATP concentration was measured using an ATP assay kit. (F) Extracellular acidification rate (ECAR) was measured using a Seahorse XF96 Flux Analyzer. (G) MCF-7 cells stably expressing HRD1 were infected with a lentivirus of PFKP for 72 h, and then cell proliferation was measured using CCK-8 assays. [file 12964_2020_679_MOESM3_ESM.tif]
